# Supplementary material for: Expert Perspectives on the Effectiveness of Psychotherapy
Source: Int J Environ Res Public Health. 2023 Sep 11;20(18):6739. doi: 10.3390/ijerph20186739 (PMC10530554; doi:10.3390/ijerph20186739)
Supplement: Supplementary file 1 [file ijerph-20-06739-s001.zip › ijerph-2538809-supplementary.pdf]

Supplementary material:

Questions to the experts:

- Do experts from different relevant areas of the macro-level evaluate the differentiation of psychotherapy care, training and its effectiveness?

- Which principles and criteria do the experts use to evaluate the options and limitations of psychotherapy and psychotherapy training in the care system? To what extent are training-, profession- or organisation-specific structurings manifested in these evaluations?

- Do they evaluate the range of psychotherapy services and their publics, as well as their societal role?

- Do the experts explicitly problematize (missing) developments of rules and resources (structures) of psychotherapeutic care?

- Do they evaluate their consequences? How?

- Which (further) development possibilities and problem-solving patterns of psychotherapeutic care are named by the experts concerned and how do they evaluate them?

- Are the problem-solving patterns linked to possible interests?
